# A dynamical framework to relate perceptual variability with multisensory information processing

**DOI:** 10.1038/srep31280

**Published:** 2016-08-09

**Authors:** Bhumika Thakur, Abhishek Mukherjee, Abhijit Sen, Arpan Banerjee

**Affiliations:** 1 Institute for Plasma Research (IPR), Bhat, Gandhinagar 382428, Gujarat, India; 2 National Brain Research Centre (NBRC), NH 8, Manesar, Gurgaon 122051, Haryana, India

## Abstract

Multisensory processing involves participation of individual sensory streams, e.g., vision, audition to facilitate perception of environmental stimuli. An experimental realization of the underlying complexity is captured by the “McGurk-effect”- incongruent auditory and visual vocalization stimuli eliciting perception of illusory speech sounds. Further studies have established that time-delay between onset of auditory and visual signals (AV lag) and perturbations in the unisensory streams are key variables that modulate perception. However, as of now only few quantitative theoretical frameworks have been proposed to understand the interplay among these psychophysical variables or the neural systems level interactions that govern perceptual variability. Here, we propose a dynamic systems model consisting of the basic ingredients of any multisensory processing, two unisensory and one multisensory sub-system (nodes) as reported by several researchers. The nodes are connected such that biophysically inspired coupling parameters and time delays become key parameters of this network. We observed that zero AV lag results in maximum synchronization of constituent nodes and the degree of synchronization decreases when we have non-zero lags. The attractor states of this network can thus be interpreted as the facilitator for stabilizing specific perceptual experience. Thereby, the dynamic model presents a quantitative framework for understanding multisensory information processing.

Multisensory signals seamlessly enrich our knowledge of the world^1^. For example, while driving a car or attending to a vocalizer in a noisy background we cannot rely on only one sensory modality be it visual or auditory, rather a harmonious interaction of visual and somatosensory or visual and auditory systems is required. In recent years, multisensory nature of perception has been the focus of much behavioral and neuroscientific research^2^. Merging information from different senses confers distinct behavioral advantages, for example, identification of audio-visual (AV) objects is more rapid^3^ than with unimodal stimuli^4^,^5^, especially when the signals are ambiguous^6^,^7^. To realize these advantages, the brain continually coordinates sensory inputs across the audiovisual^8^,^9^, visual-tactile^10^,^11^ and audio-somatic^12^ domains and combines them into coherent perceptual objects. However, operational principles of how environmental and neural variables modulate multisensory processing underlying perception are poorly understood^13^. The goal of this paper is to propose a theoretical framework that explains the empirical observations from a paradigmatic framework widely acknowledged to be an entry point in studying multisensory processes.

McGurk and MacDonald^14^ demonstrated that the sound of articulating /pa when superimposed on a video of lip movement during articulation of /ka, resulted in an illusory experience of /ta for the perceiver–a phenomenon now known as the McGurk-illusion/effect. Similar triads were also reported such as auditory /ba and visual /ga resulted in perception of /da. Several researchers have used this paradigm to study the key behavioral and neural variables that can modulate perception^15^,^16^,^17^. Ambiguity in one of the sensory streams arising from noisy stimuli affects the neuronal processing of multisensory stimuli^15^. For example, adding white noise to auditory stimuli resulted in an enhanced functional connectivity between the visual cortex and the posterior superior temporal sulcus (pSTS), a brain area populated with multisensory neurons^15^. On the other hand, an increase in ambiguity of visual stimuli (e.g., blurry video) resulted in an enhanced functional connectivity between auditory cortex and pSTS. The relative timing of different sensory input signals is another important factor in multisensory information processing^16^. Temporal proximity is a critical determinant for cross-modal integration by multisensory neurons^2^,^18^. For instance, the audiovisual integration of speech breaks down if the asynchrony between the visual lip movements and the auditory speech sounds becomes too long^16^,^19^,^20^. However a large temporal window exists over which successful integration may occur^16^,^21^,^22^,^23^. The McGurk illusion, for example persists even when the visual information leads (by up to 240 ms), or lags (by up to 60 ms) the auditory input^16^. In fact, Munhall and colleagues reported the peak of illusory response happens when the visual stimuli leads the auditory stimuli by 180–200 s^16^.

One important component not much explored in the literature is that of an integrative framework/ model that can elucidate the dynamic interactions among environmental and neural variables underlying multisensory processing of stimuli that shapes perception. The existing computational modeling literature address the spatial and temporal integration of incoming multisensory stimuli either at behavioral level or at the neural level^24^,^25^,^26^,^27^,^28^,^29^,^30^. Typically most models have 3 components, two unisensory and one multisensory module and attempt to address the integration mechanisms at the level of single units and populations at the neural level (inspired by the presence of multisensory neurons)^31^ for constructing a model. Nonetheless, the modeling approaches can be broadly classified into primarily two classes. A Bayesian framework was used by few researchers to explain how unisensory streams of audition and vision can integrate to facilitate perception by solving the spatial localization problem^24^,^25^. The second approach uses dynamic models of underlying neural systems that can be further subdivided in two classes. The first is biologically inspired modelling of neural dynamics to understand the role of predictive coding^26^ as well as spatial and temporal aspects of multisensory processing^27^,^30^ (for a review on neurocomputational approaches to modelling multisensory integration in the brain, see Ursino *et al*.^32^). The second is that of using minimal models with least amount of parameters and variables to identify the key variables affecting dynamic changes in behavior, e.g., acoustic parameters such as gap duration facilitate phonemic categorization^33^. For non-speech sounds and multisensory processing, a dynamic model was introduced by Dhamala and colleagues^23^ that explained the phenomenon of drift when slightly asynchronous audio-visual stimuli are presented. The current study incorporates the key environmental variables affecting the McGurk paradigm in a minimal model to understand their relationships with neurally relevant parameters such as the connectivity between the unisensory and multisensory systems and their potential role in oscillatory brain dynamics.

Electrophysiological signals can be conceptualized into patterned oscillations and decomposed into five frequency bands that are physiologically meaningful, delta (0.5–3.5 Hz), theta (4–7 Hz), alpha (8–12 Hz), beta (13–30 Hz) and gamma (>30 Hz)^34^. Beta band power is enhanced in fronto-parietal areas in trials where subjects perceive McGurk illusion accompanied by a reduction in theta power^17^. In general, beta band synchronization has been associated with audio-visual stimulus perception^35^. Phase synchrony and phase modulation of oscillations across the different frequency bands have been suggested to play a key role in the organization of cortical networks engaged in complex cognitive functions such as speech processing^36^ and constitute a critical component of auditory-articulatory alignment^37^. There is mounting evidence that coherence of oscillatory neural signals across cortical areas might be a crucial mechanism involved in multisensory processing^34^,^38^,^39^,^40^. Theta rhythms have been particularly associated with auditory perception of syllabic speech^41^. A recent article emphasizes the role of coherence between lip movement and brain oscillations at low frequency for intelligibility of speech stimuli^42^. Nonetheless, how perceptual categorization parametrically varies with the window of temporal integration and how oscillatory cortical activity observed by EEG/ MEG studies using McGurk-paradigm^3^,^17^,^43^ relates to perception is unclear.

Developed in 1970’s, the Kuramoto model^44^ of coupled phase oscillators is commonly used for contructing theoretical models of neurobiological networks with oscillatory dynamics^45^,^46^. In this model, the phase of an oscillation exhibited by any node of a network becomes the key variable of interest, affected by free parameters such as coupling/ connectivity terms. Thus, each Kuramoto oscillator can depict the oscillatory state of a sub-network (e. g., sections of cortical columns), captured by their circular phase alone and the overall synchronization states can capture the collective dynamics of the network. A network of Kuramoto phase oscillators provides a dynamic framework to explain the functional connectivity changes in the brain electromagnetic data^47^. The original Kuramoto model and its extensions have been used to explore mechanisms underlying oscillations in the human cortex^45^. In this article we propose a dynamical model comprising of three coupled Kuramoto oscillators, coupled via electric coupling and time-delay. Electric coupling captures physiological constraints of the audio-visual system and time-delay captures the environmental factor of temporal asynchrony (see also)^48^. Empirically such networks can be imaged non-invasively from EEG/ MEG studies^49^. Computationally, presence of time-delays enriches the attractor space of a dynamical system^50^. Our proposed model predicts the behavior of McGurk perceivers reported under ambiguous stimuli scenarios as well as during variation of AV onset lags. The rest of the paper is organized as follows. In the Methods section, we present the behavioral experimental paradigm using McGurk like stimuli, where one can study the relationship between perceptual experience and psychophysical parameters such as audio-visual onset lags. Secondly, we propose a theoretical model of multisensory perception using symmetry arguments. In the Results section we perform statistical analysis on the behavior and report the model performance under various parameter set-ups. Finally, in the Discussion section, we discuss the theoretical results in context of the experimental paradigm and argue how this modelling framework captures the key features of complex multisensory integration processes and can potentially be helpful for explaining other experimental paradigms as well.

## Methods

### Experimental paradigm

#### Subjects and stimuli

Fifty-two healthy right-handed adult participants (25 female, 27 male) of age range 20−35 years (mean age = 24.5 years, SD = 3.12) participated in a behavioral study of duration of about 45 minutes. The undertaken study design was approved by Institutional Human Ethics Committee (IHEC), National Brain Research Centre (NBRC) and the study was carried out in accordance with the guidelines set by IHEC, NBRC and in strict adherence to the declaration of Helsinki. All participants provided written informed consent in a format approved by IHEC, NBRC and reported normal vision and hearing and no history of neurological disorders. 7 video stimuli, each of 2 seconds(s) duration were prepared, of which 6 were incongruent audio-visual objects where audio recordings of a human speaker vocalizing /pa is dubbed on the lip movement of vocalization /ka (/pa-/ka) and 1 was a congruent audio-visual object /ta (/ta-/ta). The gap in onset of auditory and visual streams was varied from −300 to 450 ms in steps of 150 ms in the 6 incongruent videos. Negative sign implies that auditory stimulus onset preceded that of lip movement onset and positive implies that lip movement starts before the sound. An asymmetric range of AV lags was chosen for incongruent trials, because a previous study by Munhall and colleagues^16^ reported that the dominance of illusory perception was skewed towards positive lags where the start of lip movement precede sound onset. The congruent /ta-/ta video had synchronous onset of AV stimuli. The male speaker’s lips were in a neutral closed position during when not engaged in utterance of the syllables /ka or /ta and the articulation always started from a neutral position. Videos were created/ edited using VideoPad video-editing software (NCH Software, CO) at a frame rate of 25 frames/second and a resolution of 1280×720 pixels. The auditory /pa and /ta syllables were of 0.549 s and 0.531 s duration and were edited in Audacity software (Sourceforge.net) to minimize the background noise. The audio sampling rate was 44 kHz and had a bit rate of 128 kbps. The study was done inside a 3T MRI scanner as part of a brain imaging investigation, the results of which will be presented elsewhere.

#### Task

**Figure 1:**
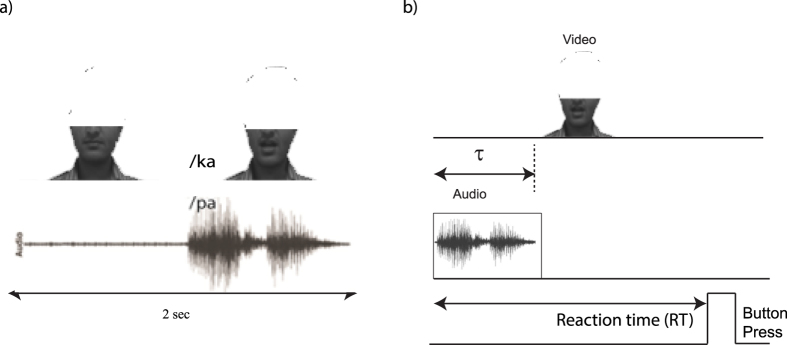
(**a**) Stimulus videos are created using visual lip movement of /ka superimposed on auditory /pa. Participants reported the auditory object they heard while watching the video using a button press response box. b) Stimuli videos were created at different audio-visual lags *τ*, the timing difference between the onset of sound and lip movement, with values ranging from [−300, 450] ms.

The task design is illustrated in Fig. 1. Inside the MR scanner, the stimuli were presented in a block design with 20 s activation blocks consisting of 10 videos of one kind of AV lag. In total there were 28 activation blocks in the whole experiment inclusive of 4 activation blocks for each stimuli category (AV lag). There were alternating 28 resting blocks, each of 20 s duration. The order of presentation of activation blocks was randomized and the same kind of block never appeared consecutively. Within a block the trial videos comprised of one kind of lag, and this is a limitation of the fMRI block design. AV stimuli were presented through a INVIVO MR - compatible CRT screen attached to the head-coil and MRI-compatible headphones (Philips, The Netherlands). Presentation software (Neurobehavioral Systems, CA) was used to display the stimuli. Participants were presented with the stimuli (Fig. 1) and asked to indicate their response based on their perception via three buttons designated for /ta, /pa and for “any other” perceptual categories. They were instructed to attend to the audio-visual stimuli and watch the speaker at all times. A fibre-optic button-pad by Curdes (Current Designs, PA, USA) was used to record the responses of the participants.

### Theoretical framework

Multisensory systems research has been built on analogies drawn from constituent unisensory processing modules. For example multisensory processing of audio-visual inputs was studied in comparison to standalone auditory and visual processing^3^,^31^,^51^. However, increasing evidences suggest that a network of unisensory and multisensory systems may be involved in multisensory task processing^15^,^17^,^23^. Thus, it is reasonable to assume that the most elemental model of multisensory perception will involve at least two unisensory systems and one multisensory system^11^,^24^,^26^,^27^ even though a recent hypothesis suggests two unisensory streams can in principle give rise to multisensory effects^13^. Furthermore, changes in oscillatory brain rhythms such as pre-stimulus enhancement of beta power and post-stimulus depreciation of theta band power have been identifed as the hallmarks of illusory perception in pre-stimuli or post stimuli regimes^17^,^52^ respectively.

As discussed in the Introduction, Kuramoto oscillators^44^,^53^ are increasingly becoming a handy tool to model oscillatory brain dynamics. The Kuramoto model was originally developed as a system of phase oscillators, each rotating at different intrinsic frequencies and coupled through the sine of their phase differences. Since then the model and its modifications have been used to study a number of biological and other phenomena such as chorusing crickets, flashing fireflies, pacemaker cells in heart. In the Kuramoto model, the phases of the individual oscillators evolving over time obey the following equations


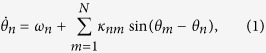


where *θ*_*n*_ is the phase and *ω*_*n*_ is the intrinsic frequency of the *n*^*th*^ oscillator and *κ*_*nm*_ is the strength of the coupling between the *n*^*th*^ and *m*^*th*^ oscillator. Here the over-dot over *θ*_*n*_ represents the time derivative.

**Figure 2:**
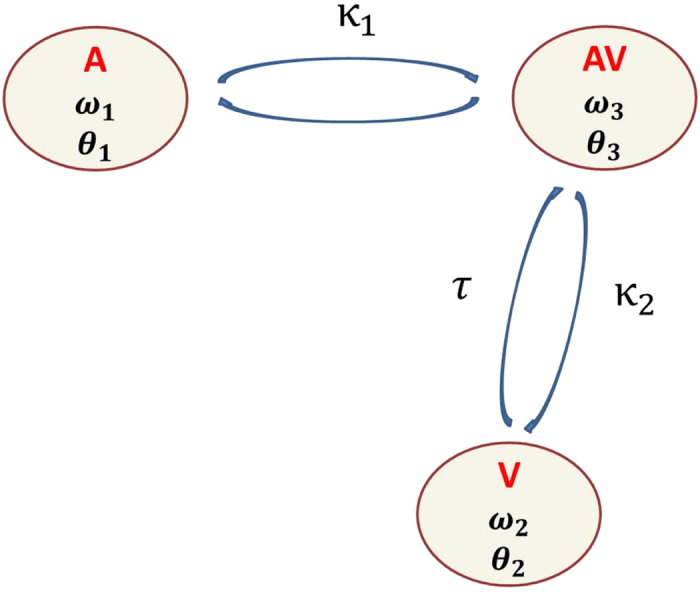
The dynamical model for multisensory speech perception consisting of three Kuramoto oscillators for positive AV lags when visual leads auditory stimulus. Oscillators A, V and AV represent the audio, visual and multisensory systems respectively. Their corresponding intrinsic frequencies are *ω*_1_, *ω*_2_ and *ω*_3_, and the respective phases are *θ*_1_, *θ*_2_ and *θ*_3_. The coupling between A and AV is instantaneous and its strength is *κ*_1_ whereas the coupling between V and AV is time delayed by the parameter *τ* and its strength is *κ*_2_. There is no direct coupling between oscillators A and V.

We consider a system of three coupled Kuramoto phase oscillators configured in the manner shown in Fig. 2. The phase of oscillator A representing the auditory system is *θ*_1_, the phase of the oscillator V representing the visual system is *θ*_2_ and the phase of the oscillator AV representing the multisensory system is *θ*_3_. Each oscillator has a distinct natural frequency of oscillation, denoted by *ω*_1_, *ω*_2_ and *ω*_3_ respectively. Thus, our model captures the functional connectivity between unisensory systems such as auditory, visual cortices and the multisensory system e.g., posterior superior temporal sulcus (pSTS) that has been observed in functional imaging studies^15^ via the coupling parameters *κ*_1_ and *κ*_2_.

Furthermore, our experimental results as well as from Munhall and colleagues^16^ indicate that a crucial parameter in creation of perceptual states is the AV lag which we capture through the time delay *τ*. In normal hearing and visual circumstances one can expect the coupling between individual sensory systems and multisensory system to be balanced. However, perturbations to one of the sensory streams such as unreliable visual or auditory signals can lead to a situation of unbalanced coupling as shown by Nath and Beauchamp^15^. There is no direct coupling between A and V oscillators in our model though in the neural system there are evidences of connectivity between A1 and V1^54^. This is primarily because we are interested to understand the key symmetries of the most simple multisensory dynamical model catered towards understanding oscillatory states of brain. Nonetheless, A and V do interact functionally in our model, since they are both coupled to AV, the functional unit of multisensory system. To model the experimental situation of AV lags (positive or negative) between the audio and visual stimuli the coupling between oscillators 2 and 3 are time delayed by the parameter *τ*. When the visual stimulus precedes the auditory stimulus (positive lag) the dynamics of phase oscillators is expressed as


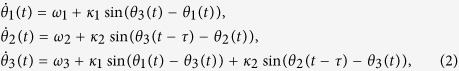


where *κ*_1_ is the strength of interaction between the auditory(A) and the AV oscillator and *κ*_2_ is the coupling strength between the visual (V) and the AV oscillator. When the auditory precedes the visual stimulus, one can consider the possibility of *τ* being negative. However, physically it does not make sense to make the present dynamics dependent on the future. Alternatively, one can intuitively relate the situation of negative *τ* to a situation where oscillator A has a time delayed coupling with AV while the visual oscillator V is instantaneously coupled. Hence, when the auditory stimulus precedes the visual stimulus, the phase oscillators can be represented as


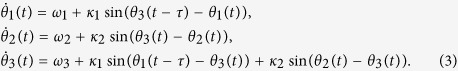


Equations 2 and 3 are similar in form but are not mirror images due to the difference in the individual frequencies of the oscillators that gives rise to asymmetry. When the oscillators are allowed to interact the frequencies of oscillations of all three units synchronize with various phase relationships (analytically derived in Results section for a simple case).

States of synchronized oscillations across a chain of phase oscillators can be mathematically identified by defining a complex order parameter, *Z*, which gives a quantitative measure of the synchronization among the oscillators and can be expressed as,


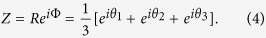


Here the amplitude *R*(*t*) is a measure of both the synchronization of the frequencies of the three oscillators as well as the phase coherence of the oscillators and Φ(*t*) measures the average phase of the system. The system can get synchronized in frequency as the coupling strengths *κ*_1_ and *κ*_2_ are increased but the oscillators can still be separated in their mutual phases while oscillating at the same frequency. For phase synchronized state a value of *R* = 1 represents maximum synchronization (0 phase difference) among the oscillators and *R* = 0 means maximally separated phases among individual oscillators.

Earlier studies have proposed that perceptual experience can be qualitatively conceptualized as attractor states of a dynamical system^23^,^33^. Stable synchronization patterns at certain relative phase relationships are attractors in a dynamical system, and instabilities signify switches in perceptual state. In this framework, the state of the order parameter or the collective variable *R* can be representative of the overall perceptual categorization and can be studied as a function of the interactions among constituent psychophysical variables, coupling strengths, the spread in the frequencies of the oscillators as well as the time delay parameter *τ*. Time delay *τ* can be interpreted as the temporal window over which integration of AV information takes place.

## Results

### Behavior

The button press responses to perceptual experience collected from each participant during the experiment were analyzed offline using customized MATLAB codes. A maximum of 40 responses were expected for each AV lag condition. Tasks with less than 35 responses were rejected since estimates of perceptual categorization may be biased during computation of percentage responses. No subjects had to be rejected based on this criteria. Behavioral responses of attempted trials of each participant were converted into percentage measures for each perceptual category, /pa, /ta or “other” corresponding to AV lags over a range [−300, 450] ms. Subsequently, 34 participants who reported perceiving /ta for at least 60% of the total responses at any AV Lag are defined as “McGurk-perceivers” and simply referred to as perceivers.

**Figure 3:**
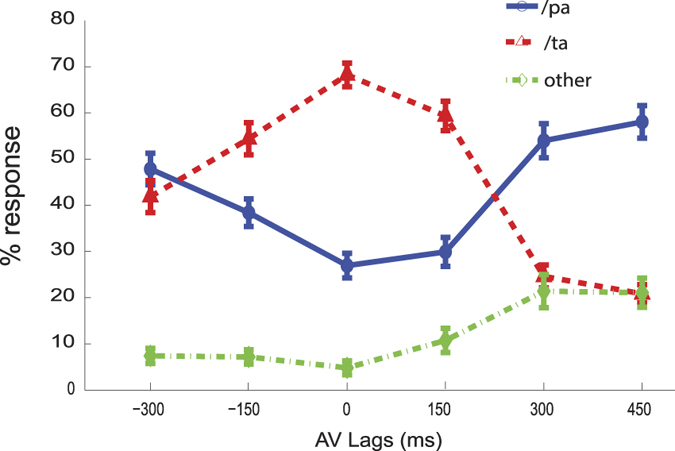
Normalized behavioral responses from 34 subjects. Mean response for each perceptual category is presented as a function of AV lag *τ*. The error bars reflect 95% significance thresholds.

We observed that perceivers report /ta maximally at an AV lag of 0 ms when the lip movement of the speaker is synchronous with the onset of auditory stimulus (Fig. 3). Conversely, /pa perception was reported the least number of times at AV lag of 0 ms. We performed an Analysis of Variance (ANOVA) over the percentage responses (without repetitions) in each category, /pa, /ta and “other” with lags and subjects treated as factors. For threshold of statistical significance set at *p* = 0.001, there was a significant change in /ta responses across lags (*F*(5, 165) = 24.46, *p* < 0.001), but not across subjects (*F*(33, 165) = 1.72, *p* = 0.0141). /pa responses also significantly varied across lags (*F*(5, 165) = 12.57, *p* < 0.001) and also across subjects (*F*(33, 165) = 5.43, *p* < 0.001). The “other” responses also significantly varied across lags (*F*(5, 165) = 7.11, *p* < 0.001) and subjects (*F*(33, 165) = 4.25, *p* < 0.001).

### Dynamical model of multisensory processing

We investigated the system of equations 2 and 3 numerically and analytically to gain insights about how time-delays may influence the stability of perceptual states. We studied the model under two scenarios, one with balanced coupling and the other with unbalanced coupling. For sake of simplicity, the analytical derivations are computed for balanced coupling conditions whereas we investigate the unbalanced coupling scenarios numerically. All numerical simulations were performed using customized MATLAB (www.mathworks.com) codes and delay-differential equation solver dde23. We were interested in the collective behavior of the system of oscillators (Fig. 2, equation 2), in particular the dynamics of the order parameter that quantified the synchronized states as a function of time-delay and coupling parameters.

#### Balanced coupling

**Figure 4:**
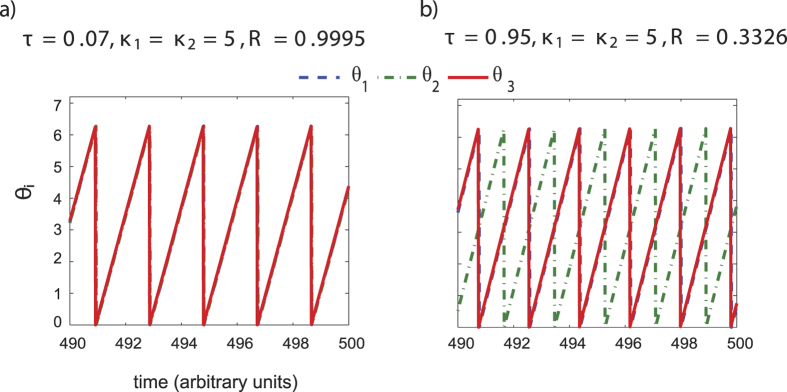
Time series of the phases *θ*_1_(*t*), *θ*_2_(*t*) and *θ*_3_(*t*) of the three Kuramoto oscillators having intrinsic frequencies *ω*_1_ = 3, *ω*_2_ = 4 and *ω*_3_ = 5 respectively (**a**) at delay *τ* = 0.07 and coupling strength *κ*_1_ = *κ*_2_ = 5. At this value of delay, all three oscillators are almost synchronized in-phase and hence the order parameter *R* = 0.9995. (**b**) at delay *τ* = 0.95 and coupling strength *κ*_1_ = *κ*_2_ = 5. At this value of delay, auditory A and multisensory AV oscillators are oscillating almost in-phase whereas the visual oscillator V is out-of phase with the other two. Hence the order parameter *R* = 0.3326

**Figure 5:**
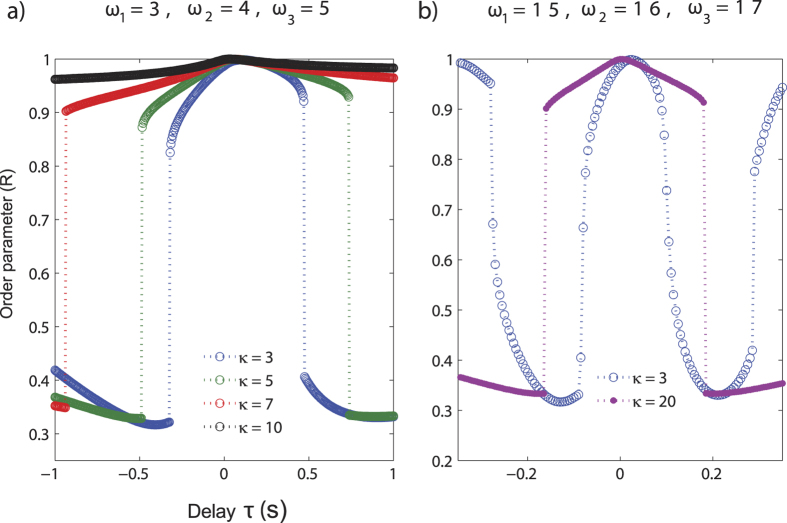
Variation of the order parameter (*R*) of the system of oscillators with delay (*τ*) for the case of balanced coupling *κ*_1_ = *κ*_2_ = *κ* when the intrinsic frequencies are a) *ω*_1_ = 3, *ω*_2_ = 4 and *ω*_3_ = 5 and b) *ω*_1_ = 15, *ω*_2_ = 16 and *ω*_3_ = 17. We have obtained the above curves for various *κ* values for the same set of initial phases.

We solve the system of equations 2 and 3 for a range of *κ* = *κ*_1_ = *κ*_2_ values from 3–20 and for different values of the time delay (*τ*). In Fig. 4 we have plotted the time series of the phases *θ*_1_, *θ*_2_ and *θ*_3_ at two delay values when coupling strength *κ* = 5. At delay *τ* = 0.07, all three oscillators are almost synchronized in-phase and hence the order parameter *R* = 0.9995. However at delay *τ* = 0.95, auditory A and multisensory AV oscillators are oscillating almost in-phase whereas the visual oscillator V is out-of phase and the order parameter *R* = 0.3326. Hence, the transition from almost complete synchronization to a lower degree of synchronization occurs at a critical value of *τ*. To explore the parameter space further, we computed the order parameter for different time delays (*τ*) and coupling strengths (*κ*) (Fig. 5). The right halves of the curves correspond to the “positive” audio-visual lags (where the visual stimulus precedes the auditory stimulus) and are obtained from the numerical integration of equation 2. The left halves of the curves correspond to “negative” audio-visual lags (where the auditory stimulus precedes the visual stimulus) and are obtained numerically from equation 3. In Fig. 5a, the initial intrinsic frequencies were set at *ω*_1_ = 3, *ω*_2_ = 4, *ω*_3_ = 5. This was roughly in *θ* frequency range (3–5 Hz) which was earlier shown to be relevant for the existence of perceptual states^17^. The initial conditions for simulations were randomized to span all possible relative phase states. For weak coupling *κ* < 1, the existence of synchronization states is not possible consistently and *R* oscillates with time. For *κ* ≥ 1, a clear synchronization state appears at least for a range of *τ* values. Two stable states of order parameter emerge, one synchronization state around *R* ≈ 1, and the other around *R* ≈ 0.33. Furthermore, we also observed that the critical value of *τ* increases with increase in *κ*. In other words the island of synchronization expands with increasing *τ*. For example for *κ* = 10 a transition happens for a high value of *τ* (not shown in Fig. 4), that is not behaviourally relevant. On the other hand, we also observed that higher intrinsic frequencies of the oscillators can recede the island of synchronization. For example, if the intrinsic frequencies are set to *ω*_1_ = 15, *ω*_2_ = 16, *ω*_3_ = 17 (beta band frequency) we see the transition from synchronization state occurs at lower values of *τ*, and the extent of synchronization regime decreases (Fig. 5b). An important point to note here is that the critical transition value of *τ* may not be same for “positive” and “negative” audio-visual lags. This stems from the disparity in the intrinsic frequencies of the oscillators.

#### Multistability and analytical solutions

**Figure 6:**
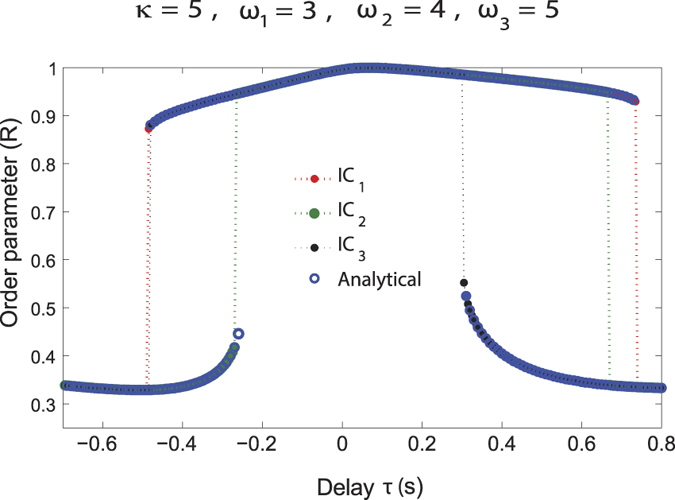
Order parameter (*R*) as a function of time delay (*τ*) for *ω*_1_ = 3, *ω*_2_ = 4, *ω*_3_ = 5 and coupling strengths *κ*_1_ = *κ*_2_ = *κ* = 5 computed using the analytical expression Eq. 15 (blue circles). Red curve is obtained by numerically integrating the model equations (Eqs 2 and 3) to get the individual phases and then using the order parameter expression (Eq. 15) for one set of initial conditions IC_1_. The green and black dash curves are obtained numerically for different sets of initial phases IC_2_ and IC_3_ respectively. We see that the system is multistable, i.e., the order parameter can take different values for same *τ* on changing initial conditions. The analytical results are in great agreement with the numerical values of the order parameter.

Multistable states of a dynamical system can be relevant for describing perception and action, in particular the inter-trial and inter-subject variability. We investigated the existence of such states in our dynamical model. The presence of a time delayed coupling provides for a rich collective dynamics of the system including the existence of multiple collective frequencies and hence the possibility of coexistence of some of these states. We have looked at this possibility by theoretical and numerical investigation of the model equations over a wide range of parameter values and randomized initial conditions in Figs 5 and 6. The system is multi-stable, meaning order parameter can take different values at same *τ* based on initial conditions (Fig. 6). The multistability seen in this model system may be related to the multistability seen in speech perception in terms of responses registered as /pa and “others” that occur 50% and approximately 10% of time respectively (Fig. 3).

The synchronized state (where all the oscillators oscillate with same frequency Ω) is given by *θ*_*i*_(*t*) = Ω*t* + *ψ*_*i*_. Let the phase shift between auditory and AV oscillator be *θ*_3_ − *θ*_1_ = *ψ*_3_ − *ψ*_1_ = *ϕ*_1_ and the phase shift between visual and AV oscillator is *θ*_3_ − *θ*_2_ = *ψ*_3_ − *ψ*_2_ = *ϕ*_2_. This gives *θ*_2_(*t* − *τ*) − *θ*_3_(*t*) = −*ϕ*_2_ − Ω*τ* and *θ*_3_(*t* − *τ*) − *θ*_2_(*t*) = *ϕ*_2_ − Ω*τ*. Using these expressions, system of equations 2 can then be written as


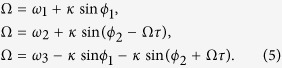


Summing across the sub-equations of Eq. 5, we obtain


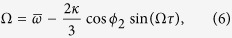


where 
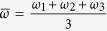
. Substituting Ω from Eq. 6 in Eq. 5, we obtain 
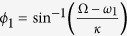
 and 
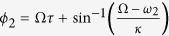
. Eq. 6 gives the collective frequency Ω as a function of delay *τ* when we have “positive” audio-visual lags. Since it is a transcendental equation, therefore we get multiple values of synchronization frequency Ω as the solution of Eq. 6 for a given value of delay. To determine which of these corresponds to a stable synchronization state, one has to do a stability analysis.

To obtain the expression of collective frequencies for the “negative” audio-visual lags, one has to do the same analysis as above using Eq. 3. For “negative” audio-visual lags, we have


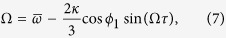


where 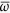 is same as above but 
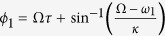
 and 
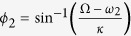
.

#### Derivation of stability condition

We perform a linear stability analysis to determine the local stability of the synchronization state by adding a small perturbation


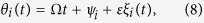


where 0 < *ε* <  < 1. Taking *ψ*_3_ − *ψ*_1_ = *ϕ*_1_ and *ψ*_3_ − *ψ*_2_ = *ϕ*_2_ gives


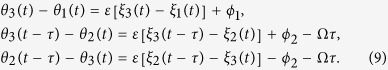


Substituting these in equations 2 we obtain


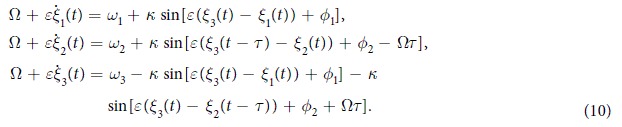


Linearizing the set of equations 10 and using 5 we obtain


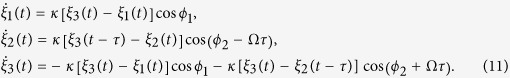


Taking *ξ*_*i*_(*t*) = *ν*_*i*_*e*^*λt*^ and substituting in the system of linear equations 11, where *λ* is the set of eigenvalues and *ν*_*i*_ are the corresponding eigenvectors, we obtain the characteristic polynomial


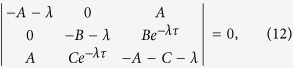


where *A* = *κ* cos*ϕ*_1_, *B* = *κ* cos(*ϕ*_2_ − Ω*τ*) and *C* = *κ* cos(*ϕ*_2_ + Ω*τ*). The stability of the synchronization frequency Ω for a given value of *κ* and *τ* can be checked using the above equation by looking at the signs of the eigenvalues *λ*. The frequency synchronized state with frequency Ω is stable if none of the eigenvalues have positive real parts.

#### Analytical expression for order parameter

The order parameter of our system is defined as


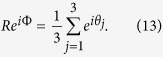


After a little algebra, we can express *R* in the form


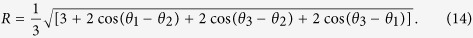


For a frequency synchronized state, *θ*_*i*_(*t*) = Ω*t* + *ψ*_*i*_ and we have taken *θ*_3_ − *θ*_1_ = *ψ*_3_ − *ψ*_1_ = *ϕ*_1_ and *θ*_3_ − *θ*_2_ = *ψ*_3_ − *ψ*_2_ = *ϕ*_2_. Therefore the analytical expression for the variation of the order parameter *R* is given by


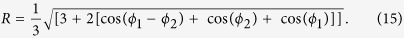


For an in-phase synchronized state *ψ*_1_ = *ψ*_2_ = *ψ*_3_. Therefore, *ϕ*_1_ = *ψ*_3_ − *ψ*_1_ = 0 and *ϕ*_2_ = *ψ*_3_ − *ψ*_2_ = 0 and hence *R* = 1. We already have the analytical expressions for *ϕ*_1_ and *ϕ*_2_: 
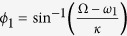
 and 
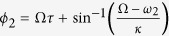
. For a given set of *κ*, *τ* and intrinsic frequencies, the synchronization frequency Ω is obtained from the transcendental equation Eq. 6 by a numerical root finding method and the stability of the corresponding state is then checked using Eq. 12. Ω values corresponding to the stable states are substituted in the expressions of *ϕ*_1_ and *ϕ*_2_ and the values of the order parameter *R* are then obtained from Eq. 15. For “negative” audio-visual lags, *R* follows the same Eq. 15 but here Ω is given by Eq. 7, 
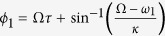
 and 
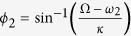
.

In Fig. 6, we have plotted the analytical as well as the numerical values of the order parameter (*R*) at corresponding values of time delay (*τ*) for the system of oscillators having intrinsic frequencies *ω*_1_ = 3, *ω*_2_ = 4, *ω*_3_ = 5 and coupling strength *κ*_1_ = *κ*_2_ = *κ* = 5 for different initial conditions. The order parameter *R* can have more than one value at a given value of delay giving rise to multistability and hysteresis. Figure 6 shows that the analytical results are in excellent agreement with the numerical values of the order parameter. The analytical expression for *R* shown in Eq. 15 holds when all the oscillators are frequency synchronized and oscillating with the synchronization frequency Ω.

#### Unbalanced coupling

**Figure 7:**
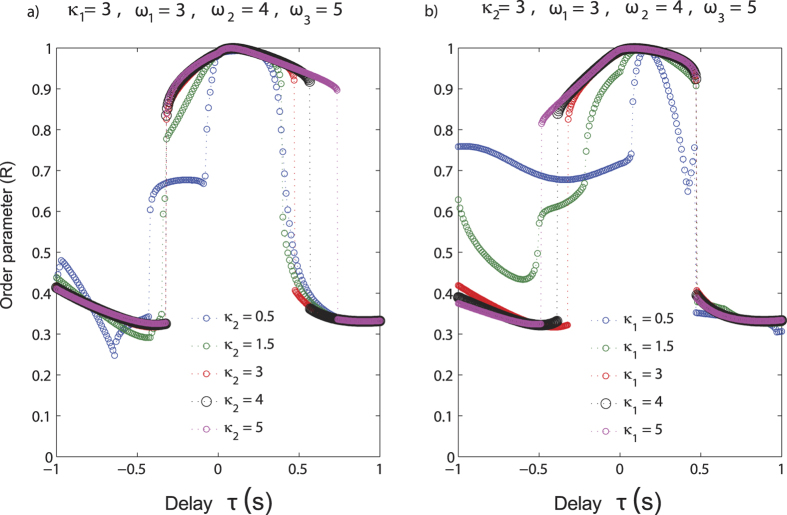
Variation of the order parameter (*R*) with delay (*τ*) of the system of oscillators having intrinsic frequencies *ω*_1_ = 3, *ω*_2_ = 4 and *ω*_3_ = 5 (**a**) when the coupling strength between A and AV, (*κ*_1_) is kept constant at 3 and the coupling between V and AV, (*κ*_2_) is varied from 0.5 to 5. (**b**) the coupling strength between V and AV, (*κ*_2_) is kept constant at 3 and the coupling between A and AV, (*κ*_1_) is varied from 0.5 to 5.

We have investigated the unbalanced coupling scenarios following two routes. First, we have kept *κ*_1_ fixed at the value 3 and varied *κ*_2_ between [0.5, 5] (Fig. 7a). *κ*_2_ < *κ*_1_, is equivalent to the situation where ambiguity is introduced in visual stream because of which the coupling changes, e.g. in the Nath and Beauchamp study^15^. *κ*_2_ > *κ*_1_ is the situation where visual coupling is increased, auditory being unreliable. In both cases existence of a synchronization state and non-synchronous state is possible as long as *κ*_1_ ≥ 1. When *κ*_1_ is fixed the island of synchronization increases with increasing values of *κ*_2_ indicating, that time-delayed interactions with the visual-multimodal system is crucial for perception. For negative AV lags, e.g., when auditory precedes the visual stimulus no drastic change in critical delay value occurs when *κ*_1_ is unchanged.

Second, we vary *κ*_1_ while keeping *κ*_2_ constant. Here we see that the critical delay value for transition from phase-synchronous to non-synchronous states shifts such that the regime of synchronization increases for negative AV lags corresponding to the scenario where auditory stimulus precedes the visual stimulus (Fig. 7b). Multistable states are possible and the possibility of existence of two states, one synchronous and one non-synchronous is critically dependent on *κ*_1_ (Fig. 7b).

Overall we find time delays and coupling strength facilitate the existence of synchronous states and the intrinsic frequency of oscillations reduces the temporal window over which AV signals can integrate. Thus, the synchronization window can extend over an entire range of AV lags. This is in qualitative agreement with the trend seen for the McGurk stimuli (Fig. 3). Existence of multistability is demonstrated in Fig. 6 explicitly but also observed for the coupling scenarios discussed in Fig. 5 and Fig. 7. Small amounts of perturbations from numerical instabilities was sufficient to generate transitions between the stable states of the order parameter.

## Discussion

The main purpose of this work is to conceptualize the observations from an experimental paradigm, that has been over the years a bedrock to study multisensory information processing, with a simple dynamical model to illustrate the role of environmental variables and connectivity topologies between neural subsystems in shaping of perceptual states. The Kuramoto oscillator framework provides the added advantage of linking the perceptual states to simultaneously observed neuronal oscillations from EEG/ MEG data. The perceptual dynamics observed for /ta response by Munhall and colleagues^16^ and our behavioral recordings (Fig. 3) are in complete agreement with the order parameter dynamics observed from our model (Figs 5,6 and 7). We see that the illusory perception is reported maximally within a range of lags [−150, 300] ms. Beyond this range, the auditory /pa response dominates, for both positive and negative AV lags. We have considered a system of three phase oscillators, each representing the auditory, visual and multisensory systems. Superior colliculus (SC) and posterior superior temporal sulcus (pSTS) are two structures at the sub-cortical and cortical level respectively that can get representation in the multisensory system. SC is known to have bimodal neurons that can receive inputs from both the auditory and visual systems^55^. Similarly, pSTS has been established by several researchers as the locus of cortical processing of multisensory integration^15^,^56^,^57^,^58^,^59^. Thus a network comprising of auditory, visual areas and pSTS (with contributions from SC) can form the most elemental network for multisensory processing. Experimental validation about the presence of such networks have been provided by earlier studies such as Nath and Beauchamp^15^ using a fMRI paradigm. An important parameter in our model is the coupling between AV - A and AV - V oscillators. Careful design of such coupling parameters motivated from experimental studies can capture how the biophysically realistic symmetry that exists in the underlying neural system can influence behavior^48^. Broadly, the multisensory functional unit can represent qualitatively the composite contributions of all structures such as superior colliculus, pSTS and other areas. Detailed modelling of each region along with the coupling observed in experimental fMRI data is possible, but the results from such descriptions are hard to generalize and fail to capture the overarching symmetry. Due to similar reasons we chose to ignore the possible coupling between A and V resulting from direct anatomical connections between A1 and V1 areas^54^,^60^,^61^ in our model. In our model the signals from A and V are conveyed to the multisensory oscillator (AV) where information from the two modalities are integrated. The feedback connections from the pSTS to the unimodal areas are directly considered in the form of a bidirectional coupling between pSTS and auditory and visual areas. Nevertheless, the weighted contributions of direct coupling between A1-V1 and their interplay with the coupling between multisensory to unisensory areas can be studied in the dynamical framework. In future, a corresponding behavioral paradigm needs to be developed to test the predictions of such a model.

The coupling parameter in our model helps in two ways, first in the explanation of the existence and destabilization of perceptual states upon variation of time delays and second to provide a link between observed perceptual dynamics and neuronal oscillations. It is well-known that environmental demands modulate the effective connectivity dynamics among individual sensory systems and multisensory system^15^. The information from the more reliable modality is given a stronger weight^62^. For the McGurk paradigm, Nath and colleagues^15^ found that the STS was connected more strongly to a sensory cortex when the corresponding sensory modality was reliable (less noisy). In our model, *κ*_1_>*κ*_2_ may correspond to a scenario of increased functional connectivity between the STS and the auditory cortex when the auditory modality is more reliable and *κ*_2_>*κ*_1_ can be realized as increased functional connectivity between the STS and visual cortex when the visual modality is more reliable. Since, there are both top-down and bottom-up connections throughout the cortical processing hierarchy^63^,^64^, we have incorporated bidirectional connections into our dynamical model. Importantly, for unbalanced coupling scenarios also the synchronized states exist indicating illusory experience is possible in presence of noisy stimuli. Electrophysiological studies indicate that a pre-stimulus theta band activity primes the network for multisensory information processing towards illusory perceptual categorization^17^. Our results indicate how the intrinsic frequencies of oscillators set at theta regime can support the perceptual dynamics as observed by varying the AV lag in the multisensory stimulus (compare Fig. 5 to Fig. 3). We predict that tuning of oscillators at higher frequency band may shorten the window of temporal integration. In other words narrower window of temporal integration will require phase synchrony at higher frequency such as beta and gamma. Interestingly our results suggest that coupling strength has to be relatively high if temporal integration is facilitated by beta band synchrony to produce the cross-modal percepts for a wider value of AV lags (Fig. 5b). This is in-line with the proposition of Luo and Poeppel^41^, who proposed a low and high frequency segregation of auditory processing. From our study we can predict that low frequency processing (e.g. syllabic speech) may be carried out by relatively weakly coupled unisensory and multisensory systems whereas high frequency processing e.g., diphonic speech/ tones require more stronger connectivity strengths among sensory systems. In summary, the minimal model of multisensory integration is geared towards linking the symmetries in neuronal connectivity and dynamics with environmental demands and behavior.

The existing models of multisensory integration are mostly based on Bayesian framework and neural networks. These studies consider different kinds of interactions among multisensory and unisensory areas. One category of models considers that the unisensory stimuli are processed separately in the primary cortices, without a significant cross-modal interaction, and multisensory integration take place in higher associative cortical areas such as SC via feedforward convergence from multiple unimodal areas^65^. Second category of models assumes only direct lateral connections between the two unimodal areas^29^,^30^ and excludes the involvement of multisensory regions. They argue that the direct connections among early processing areas (modality-specific areas such as visual and auditory) play a pivotal role in multisensory integration. These models are based on recent anatomical tracing studies in monkeys (macaque) and human subjects that have shown direct connections between auditory and visual areas including primary cortices (V1 and A1)^54^,^60^. Similar studies have also reported projections to/from somatosensory cortex from/to auditory and visual areas^66^. The heteromodal connections between the primary somatosensory cortex and the primary auditory cortex were also reported in the gerbils^67^. In marmosets, projections from the retroinsular area of the somatosensory cortex to the caudiomedial belt auditory area were also observed^63^ in line with a similar observation in the Old World monkey^68^. Third category of models excludes direct connections between the unimodal areas but considers both a feedforward connection to a multisensory area, and a feedback from the multisensory area to the unisensory ones^28^. Finally, the fourth category of models incorporates all previous connections together, i.e., the feeback and feedforward connections between unimodal and multimodal systems along with the direct connections between the unimodal systems^11^,^69^. Most of these studies do not consider the processing of temporal relationships among the unimodal stimulus components, e.g., AV lags^16^, gap duration in speech perceptual categorization^33^. Our dynamical model (which comes under the third category) comprising of three Kuramoto oscillators attempts to do so in a minimalist manner. An earlier attempt in this direction was made by Dhamala and colleagues^23^ who modelled the rhythmic multisensory paradigm in the audio-visual domain by considering the phase dynamics of two interacting periodic oscillators and investigated the behavioral effects of relative timings of different sensory signals. In their rhythmic paradigm, temporally congruent multisensory stimuli were expected to cause a percept of synchrony. On the other hand, incongruent stimuli could cause a percept of asynchrony or another possible state, the non-phase locked state (drift or neutral percept) because it represented failure in multisensory integration. The non-phase locked state was qualitatively different from the percept of asynchrony. Dhamala and colleagues then used fMRI and simultaneous behavioral recordings to confirm the involvement of a distributed brain network for the multisensory processing of periodic auditory-visual stimuli. The study proposed that the solution space of a hypothetical system of two coupled oscillators corresponded to the perceptual solution space of human multisensory integration and to selected activation of brain regions. Though the authors propose that their results indicate definite involvement of superior colliculus in the perception of synchrony, they have not captured the dynamics of multisensory system as a functional unit directly in the theoretical model, rather emphasizing on the periodicity as the source of categorization. Nonetheless, the rhythmic paradigm is somewhat unrealistic in the context of multisensory speech stimuli. Hence, a broader dynamical framework for multisensory perception is warranted. A key difference between our model and that of Dhamala *et al*.^23^ is that they have not incorporated the time lag or asynchrony between the audio and visual stimuli explicitly in their theoretical model. In our model we capture the AV lag by introducing a time delay *τ* between the multisensory (AV) and unisensory (V/A) oscillators and study the variation in perceptual stability as a function of this parameter. Subsequently, we propose that the order parameter or the collective variable R of the oscillator system can be representative of overall perceptual categorization.

Multistability is a key aspect of perceptual behavior and in general biological systems^70^. In the McGurk paradigm, presence of responses registered as /ka (the visual stimuli) or fused percepts such as /pa-ka in addition to illusory /ta or auditory /pa can be conceptualized within the framework of multistability. We propose that the different responses registered as /pa, /ta and “others” in our behavioral data can be related to the multistability in the final dynamical state of our coupled oscillator system. The advantage of a dynamical systems approach is that the presence of multistability can be very elegantly explained^71^. Detailed analysis of multistability can be extended to psychophysics of speech perception studies^33^ in future as well as with other paradigms. As we have shown in the Results section, the presence of time delay makes our dynamical system highly multi-stable. For different choices of initial conditions, the system can go to different stable synchronization states. We propose that the multistability in the final state of the dynamical system can be related to the variation observed in the responses of the participants to same stimuli.

The significant achievement of our model is that it captures the key features of complex multisensory integration processes at the level of behavior and links the structural and functional constraints in the underlying neural systems to ongoing behavioral states. Our results show that our model successfully simulates the temporal constraints on the McGurk effect and the variation in the responses to the same stimuli. Any multisensory behavior can be broken into combinations of individual unisensory and multisensory systems. One can then map the constraints posed by the environmental variables onto the parameter space of a dynamical system and study the emerging perceptual states. For example, recent studies question the idea of multisensory awareness stemming from a mere integration of unisensory systems^13^,^72^. This is a challenge to the traditional way of looking at a multisensory task from the perspective of serial or parallel unisensory processes. Dynamical systems offer an attractive approach to test the environmental constraints on a hypothesized multisensory pathway using modelling, behavior and brain mapping tools. Here, the integration is not identified as a separate process but rather an outcome of the dynamical interactions among unisensory and multisensory neuron populations. In fact the role of factors like attention can be incorporated in the dynamical model by introducing more mathematical complexity, either by coupling parameter or introducing a separate module, and presents a scope of future work. Experimentally this will require recording and analyzing eye-tracking data to monitor and parametrize attentional factors. Nonetheless, new experimental paradigms have to be developed to eventually test the predicted outcomes from modelling approaches such as ours.

## Additional Information

**How to cite this article**: Thakur, B. *et al*. A dynamical framework to relate perceptual variability with multisensory information processing. *Sci. Rep.* **6**, 31280; doi: 10.1038/srep31280 (2016).
